# Changes in 24‐h energy expenditure, substrate oxidation, and body composition following resistance exercise and a high protein diet via whey protein supplementation in healthy older men

**DOI:** 10.14814/phy2.15268

**Published:** 2022-06-15

**Authors:** Corbin Griffen, Derek Renshaw, Michael Duncan, Martin O. Weickert, John Hattersley

**Affiliations:** ^1^ Centre for Sport, Exercise and Life Sciences Research Institute for Health and Wellbeing Coventry University Coventry UK; ^2^ 2708 Human Metabolism Research Unit University Hospitals Coventry and Warwickshire NHS Trust Coventry UK; ^3^ 2706 School of Life Sciences Faculty of Health and Life Sciences Coventry University Coventry UK; ^4^ 2708 Department of Endocrinology and Diabetes University Hospitals Coventry and Warwickshire NHS Trust Coventry UK; ^5^ Warwick Medical School University of Warwick Coventry UK; ^6^ School of Engineering University of Warwick Coventry UK

**Keywords:** aging, body composition, energy expenditure, protein, resistance exercise, substrate oxidation

## Abstract

**Purpose:**

To investigate changes in 24‐h energy expenditure (EE), substrate oxidation, and body composition following resistance exercise (RE) and a high protein diet via whey protein supplementation (alone and combined) in healthy older men.

**Methods:**

In a pooled groups analysis, 33 healthy older men [(mean ± SE) age: 67 ± 1 years; BMI: 25.4 ± 0.4 kg/m^2^] were randomized to either RE (2×/week; *n* = 17) or non‐exercise (*n *= 16) and either a high protein diet via whey protein supplementation (PRO, 2 × 25 g whey protein isolate/d; *n* = 17) or control (CON, 2 × 23.75 g maltodextrin/d; *n* = 16). An exploratory sub‐analysis was also conducted between RE+CON (*n* = 8) and RE+PRO (*n* = 9). At baseline and 12 weeks, participants resided in respiration chambers for measurement of 24‐h EE and substrate oxidation and wore an accelerometer for 7 days for estimation of free‐living EE.

**Results:**

Resistance exercise resulted in greater increases in fat‐free mass (1.0 ± 0.3 kg), resting metabolic rate [(RMR) 36 ± 14 kcal/d], sedentary EE (60 ± 33 kcal/d), and sleeping metabolic rate [(SMR) 45 ± 7 kcal/d] compared to non‐exercise (*p* < 0.05); however, RE decreased activity energy expenditure in free‐living (−90 ± 25 kcal/d; *p* = 0.049) and non‐exercise activity inside the respiration chamber (−1.9 ± 1.1%; *p* = 0.049). PRO decreased fat mass [(FM) −0.5 ± 0.3 kg], increased overnight protein oxidation (30 ± 6 g/d), and decreased 24‐h protein balance (−20 ± 4 g/d) greater than CON (*p* < 0.05). RE+PRO decreased FM (−1.0 ± 0.5 kg) greater than RE+CON (*p* = 0.04).

**Conclusion:**

Resistance exercise significantly increased RMR, SMR, and sedentary EE in healthy older men, but not total EE. PRO alone and combined with RE decreased FM and aided body weight maintenance. This study was registered at clinicaltrials.gov as NCT03299972.

## 1 INTRODUCTION

Increased fat mass (FM) and declines in fat‐free mass (FFM) characterize age‐related changes in body composition (St‐Onge, 2005). Skeletal muscle, which accounts for ~45% of FFM (Geisler & Müller, 2017), decreases by ~0.5–1% per annum after ~45 years of age (Janssen, 2010). When accompanied by concomitant reductions in muscle strength and physical function, this gives rise to a disease known as sarcopenia (Cruz‐Jentoft et al., 2019). Contrary to skeletal muscle mass (SMM), FM has been shown to increase by ~0.2% per annum from 20 years of age (Imboden et al., 2017; Westerterp, 2018a). The coexistence of sarcopenia and adiposity, termed sarcopenic obesity, is of great concern as they act synergistically, increasing the risk of metabolic and cardiovascular disease (CVD), and ultimately mortality (Wannamethee & Atkins, 2015).

Body composition changes with age are ascribed to alterations in energy balance (St‐Onge & Gallagher, 2010). Regarding the energy expenditure (EE) component of the energy balance equation (rate of energy storage = rate of energy intake (EI) − rate of EE), aging is associated with declines in all three major constituents: resting metabolic rate (RMR), which accounts for 60–80%; activity energy expenditure (AEE), which comprises ~20–50%; and diet‐induced thermogenesis (DIT), which uses 5–10% (Hall et al., 2012; Manini, 2010). The decline in RMR occurs at a rate of ~1–2% per decade from the age of 30 (Elia et al., 2000). Fat‐free mass accounts for 50–70% of the variance, of which SMM accounts for ~25% (Bosy‐Westphal et al., 2003; Gallagher et al., 1998; Geisler et al., 2016a). Hence, interventions that target skeletal muscle and curb sarcopenia may mitigate age‐related declines in components of EE, improve energy balance, and attenuate adiposity in older adults.

Resistance exercise (RE) is a potent stimulus to increase skeletal muscle and FFM and is considered the primary intervention to mitigate sarcopenia (Phillips & Martinson, 2019). Alongside beneficial effects on FFM, increases in total EE (TEE) (Hunter et al., 2000), RMR (Hunter et al., 2004) and 24‐h fat oxidation (Treuth et al., 1995), and decreases in the energetic cost of walking (Valenti et al., 2016) and FM (Westcott, 2012) have been observed in older adults following RE training. Increased EE following RE is often attributed to the energetic cost of increased skeletal muscle and FFM (Hunter et al., 2004), which have specific metabolic rates of 12.6 and 24 kcal/kg/d, respectively (Hall, 2006; Wang et al., 2010). Although, it is important to note that acute factors also contribute, namely excess post‐exercise oxygen consumption (EPOC), which includes, but is not limited to, glycogen, adenosine triphosphate (ATP) and creatine phosphate resynthesizing, protein turnover, ion redistribution, blood and muscle oxygen replenishment, and residual hormone effects (Børsheim & Bahr, 2003).

While RE has repeatedly been shown to increase aspects of 24‐h EE, a potential caveat in older individuals is the frequently reported compensatory reduction in AEE, particularly spontaneous physical activity (SPA) (Westerterp, 2018a, b). This has been postulated to occur due to training‐related fatigue (Hunter et al., 2018) and/or energy compensation to maintain energy balance (Careau et al., 2021; Hall et al., 2012). Previous work in older adults has shown that this effect may be eliminated, without any dampened effects on SMM or strength, by performing load‐matched RE twice as opposed to three times per week (Hunter et al., 2013). However, participants in this study also participated in aerobic exercise (AE), highlighting the need for further research to determine whether performing RE twice as opposed to thrice weekly without AE eliminates energy compensation in older adults.

In addition to RE, a high protein diet may also assist in attenuating sarcopenia (Phillips & Martinson, 2019). At present, protein recommendations for adults aged ≥19 years are set according to the recommended dietary allowance (RDA) of 0.8 g/kg/d (Institute of Medicine, 2005). However, working groups reason that the RDA is insufficient for older individuals to curb sarcopenia and intakes of ~1.2 g/kg/d (Bauer et al., 2013; Deutz et al., 2014) and even up to 2× the RDA of 1.6 g/kg/d (Phillips et al., 2016) should be consumed. Indeed, intakes of dietary protein towards the higher end of these recommendations (~1.4–1.6 g/kg/d) have been shown to increase FFM in older adults (Bauer et al., 2015; Bell et al., 2017; Mitchell et al., 2017; Norton et al., 2016; Park et al., 2018). Normative data in older adults, however, report protein intakes of ~1 g/kg/d (Farsijani et al., 2017), highlighting the need for increased intake to mitigate sarcopenia (Phillips & Martinson, 2019).

A high protein diet (25–30% of EI) may also mitigate age‐related reductions in EE and aid body‐weight management (Drummen et al., 2018). For example, increases in TEE, RMR, sleeping metabolic rate (SMR), and DIT (Bray et al., 2015; Drummen et al., 2020; Martens et al., 2015; Oliveira et al., 2021; Sutton et al., 2016), improved metabolic efficiency of physical activity (Apolzan et al. 2014; Martens et al., 2015), decreased 24‐h respiratory quotient (RQ) and fat balance (Drummen et al., 2020; Lejeune et al., 2006; Martens et al., 2015; Oliveira et al., 2021; Smeets et al., 2013), and an adaptive thermogenic increase in TEE and SMR when dietary protein intake is returned to baseline levels (Bray et al., 2015) have been reported following a high protein diet. Protein‐induced increase in EE may be explained by the ATP required for metabolism, including protein breakdown, synthesis and storage, and oxidation, including urea synthesis (Drummen et al., 2018). Gluconeogenesis due to a surplus of dietary protein also contributes to the increased EE (Veldhorst et al., 2009), as does protein‐induced increases in skeletal muscle and FFM (Drummen et al., 2018).

Despite the fact aforementioned studies report beneficial effects on EE following a high protein diet, the majority of studies [excluding Drummen et al. (2020)] were conducted on young adults, and some longitudinal studies in older adults have reported conflicting findings (Luger et al., 2013; Negro et al., 2019). These null findings were, however, likely due to an insufficient increase in dietary protein intake during the intervention period (≤0.1 g/kg/d). Nonetheless, although Drummen et al. (2020) reported protein‐induced effects on EE (specifically, increased RMR) in older adults (~65 years) following ~34 months of a high protein weight maintenance diet, EE was only assessed following but not prior to the intervention. Consequently, further research on the pre‐post longitudinal energetic effects of a high protein diet in older adults is needed.

Meta‐analyses indicate that increased dietary protein intake combined with RE may synergistically decrease both absolute and %FM (Liao et al., 2017) and aid muscle hypertrophy (Cermak et al., 2012; Finger et al., 2015; Liao et al., 2017; Morton et al., 2018). However, the majority of intervention studies in older adults have been unable to replicate supplemental increases in skeletal muscle or FFM (Arnarson et al., 2013; Candow et al., 2006; Chalé et al., 2013; Dulac et al., 2021; Englund et al., 2017; Gryson et al., 2014; Holm et al., 2008; Holwerda et al., 2018; Kukuljan et al., 2009; Leenders et al., 2013; Maltais et al., 2016; Ottestad et al., 2017; Shahar et al., 2013; Thomson et al., 2016; Verdijk et al., 2009; Verreijen et al., 2017). Similarly, studies investigating the combined effects on components of EE have also observed no synergistic effects (Amamou et al., 2017; Campbell et al., 1994; Maltais et al., 2016; Weinheimer et al., 2012). Null findings may be attributed to an inadequate sample size and lack of statistical power (Campbell et al., 1994), an adequate habitual protein intake of participants (Weinheimer et al., 2012), and an insufficient increase in dietary protein intake from baseline (<0.4 g/kg/d) and a total dietary protein intake of <1.6 g/kg/d during the intervention period (Amamou et al., 2017; Maltais et al., 2016). The latter of these, as suggested by others (Morton et al., 2018; Park et al., 2018), might be the breakpoints required to maximally augment increases in skeletal muscle and FFM and consequent increases in components of EE.

A limitation of the above‐cited studies investigating the synergistic effects of RE and increased dietary protein intake was the sole measurement of EE in the resting state (i.e., RMR). Twenty‐four‐hour EE is not constant and is regulated by numerous factors such as time of day and food intake (Schoffelen & Plasqui, 2018); therefore, analysis of only RMR does not provide a comprehensive analysis of the synergistic effects on 24‐h energy metabolism. Consequently, analysis of the synergistic effects on multiple components of 24‐h EE (i.e., RMR, SMR, AEE, DIT, and TEE) is warranted.

The primary aim of this study was to examine changes in 24‐h EE, substrate oxidation, and body composition in healthy older men following 12 weeks of RE and a high protein diet via whey protein supplementation [which aimed to increase dietary protein intake by ≥0.4 g/kg/d to ~1.6 g/kg/d (~25% of EI)]. A secondary aim was to conduct an exploratory sub‐analysis to determine whether RE combined with a high protein diet via whey protein supplementation synergistically increases EE and improves body composition. We hypothesized that RE and a high protein diet individually would increase components of EE, substrate oxidation and improve body weight maintenance and composition. We also postulated that there would be a synergistic effect when interventions were combined.

## 2 MATERIALS AND METHODS

### 2.1 Participants

Thirty‐three healthy, community‐dwelling older men [(mean ± SE) age: 67 ± 1 years] participated in this study. Full details of the eligibility criteria have been previously described (Griffen et al., 2022). Briefly, participants were eligible if they: were (i) aged 60–80 years; (ii) a non‐smoker; (iii) weight stable (± <3 kg change in the previous 6 months); had (iv) a BMI between 18.5 and 30 kg/m^2^; (v) not participated in RE in the previous 6 months; (vi) no past or existing history of cancer, diabetes mellitus, or cardiovascular, thyroid, or renal disease; and (vii) were not taking statins, non‐steroidal anti‐inflammatory drugs, or medication that affects metabolism. The study was approved by Coventry University Ethics Committee (project code: P59723) and was registered at clinicaltrials.gov as NCT03299972. All participants provided written informed consent prior to enrollment.

### 2.2 Design

The present study is a pooled analysis of a 12‐week randomized, controlled, double‐blind, 4‐arm (control, a high protein diet via whey protein supplementation, RE + control, RE + a high protein diet) parallel group trial which took place between October 2017 and May 2019 (Griffen et al., 2022). In the present study, RE (*n* = 17) and non‐exercise (*n *= 16) groups were pooled and compared to one another, as were high protein (PRO; *n* = 17) and control (CON; *n* = 16) groups. Additionally, an exploratory sub‐analysis was conducted between RE + control (RE+CON; *n* = 8) and RE + a high protein diet via whey protein supplementation (RE+PRO; *n* = 9) groups to determine synergistic effects. The experimental design and pooling of groups for analysis are shown diagrammatically in Figure S1. Measurements were taken at baseline and following the intervention.

### 2.3 Exercise training

Supervised whole‐body RE was performed twice weekly at Coventry University. Sessions occurred at least 48 h apart and the final session occurred >72 h prior to post‐intervention metabolic testing. Each session consisted of a 5‐min warm‐up on a cycle ergometer followed by three sets of leg press, lateral row, hamstring curl, chest press, leg extension, and shoulder press (in that order) on fixed RE machines (Life Fitness, Rosemont, Illinois, USA). During the first 4 weeks of training, exercise intensity began at 60% one‐repetition maximum [(1RM) 10–12 repetitions per set] and was gradually increased by ~5–7% per week to 80% 1RM (8 repetitions per set), where it remained until the end of the intervention. The final set of each exercise was performed to volitional failure, which was defined as the inability to perform an additional repetition with the correct form. Completion of repetitions was monitored during each session. Resting periods of 60 s and 3 min were allocated between sets and exercises, respectively. The intensity was adjusted according to 1RM tests performed every 4 weeks and when participants were able to complete >12 repetitions on the final set of each exercise. Sessions concluded with a 5‐min cool‐down on a cycle ergometer. Compliance was monitored using a training log.

### 2.4 Dietary intervention

Participants in the PRO group ingested 25 g whey protein isolate (including ~3 g leucine) (Instantized BiPRO; Agropur, Quebec, Canada), whereas participants in the CON group consumed an energy‐matched control (23.75 g maltodextrin; Myprotein, Northwich, UK) twice daily. Supplements were consumed directly after breakfast and lunch. The whey protein dosing regimen employed was chosen based on previous studies that have demonstrated that older adults typically consume insufficient amounts of dietary protein at breakfast and lunch to maximally stimulate rates of muscle protein synthesis (MPS) (~0.2 and ~0.3 g/kg, respectively) (Farsijani et al., 2017; Smeuninx et al., 2020; Tieland et al., 2012a). In addition, it was hypothesized that the whey protein dosing regimen would increase daily dietary protein intake from ~1.0 (~15–17% of EI) to ~1.6 g/kg/d (~25% of EI), the upper intake recommended to curb sarcopenia (Phillips & Martinson, 2019). Consumption of the final supplement occurred the day before (~32 h prior to) post‐intervention metabolic testing. The nutritional composition of the experimental supplements can be seen in Table S1. Supplements were unflavored, similar in powder weight, and were provided in opaque sachets in a double‐blinded manner (Flexible Packaging Services Ltd, Wirral, UK). Participants prepared their supplement beverages at home by dissolving the contents with ~200 ml of water combined with unsweetened cordial to taste. Compliance was calculated from returned wrappers and unused sachets. The effectiveness of participant blinding was assessed by a questionnaire at the end of the study.

### 2.5 Dietary intake

Participants were instructed to not alter their habitual diet for the duration of the study. Participants completed 3‐day food records (2 weekdays and 1 weekend day) at baseline (prior to commencing the intervention) and during weeks 6 and 12. Dietary records were analyzed using dietary analysis software (Nutritics Version 5.097; Nutritics, Dublin, Ireland). Participants replicated their dietary intake on the day of pre‐ and post‐intervention metabolic testing.

### 2.6 Body composition

Body composition was measured in the morning by bioelectrical impedance analysis (BIA) (BC‐418 MA; Tanita Corporation, Tokyo, Japan). Measurement occurred at the same time of day (± 1 h) and participants were asked to consume the same breakfast prior to pre‐ and post‐intervention measurements. Participants voided their bladder prior to measurement and wore minimal clothing. Skeletal muscle mass was estimated using the equation of Janssen et al. (2000).

### 2.7 Respiration chamber

**FIGURE 1:**
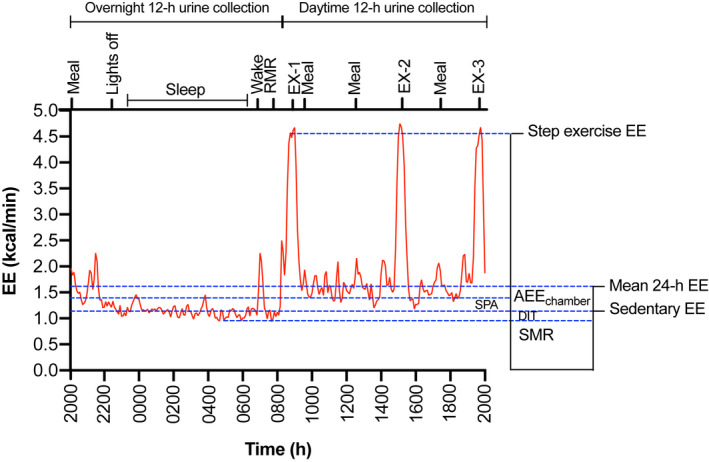
Schematic of the 24‐h respiration chamber protocol. Energy expenditure (*y*‐axis) is plotted against time (*x*‐axis bottom) for one participant (ID: 008_baseline; age: 72 years). The protocol is noted on top of the *x*‐axis. Components of 24‐h EE calculated inside the respiration chamber are illustrated with blue dashed lines. AEE_chamber_, activity energy expenditure; DIT, diet‐induced thermogenesis; EE, energy expenditure; EX‐1, step exercise bout 1 (fasted); EX‐2, step exercise bout 2; EX‐3, step exercise bout 3; SMR, sleeping metabolic rate; SPA, spontaneous physical activity

Participants resided in respiration chambers for measurement of 24‐h EE and substrate oxidation. Briefly, the respiration chamber is an airtight and thermally insulated living space (floor dimensions: 2.9 m × 2.1 m) containing a bed, desk, chair, computer, and freezer toilet (Schoffelen et al., 1997). Environmental conditions were continuously controlled (relative humidity: 57 ± 5%; temperature: 24 ± 0.5 °C). Prior to baseline testing, participants attended a familiarization session to be accustomed to the chamber environment. On experimental testing days, participants entered the respiration chamber at ~1930 h and left at 2000 h the following evening. The protocol for the 24‐h measurement period (2000–2000 h) is shown diagrammatically in Figure 1. Whilst residing inside the respiration chamber, participants were fed a study diet designed to achieve energy balance (see Table S2 for example). The diet consisted of ~45% of energy from carbohydrate, ~20% of energy from protein, and ~35% of energy from fat. The macronutrient distribution of the diet was identical for pre‐ and post‐intervention visits to determine the effects of the interventions on FFM and subsequent effects on energetics in the absence of acute protein‐induced effects on EE. Energy requirements for each participant were calculated prior to entering the respiration chamber using the Katch‐McArdle basal metabolic rate (BMR) formula (McArdle et al., 1991) multiplied by an activity factor of 1.47. Requirements were adjusted based on measured RMR within the respiration chamber the next morning (detailed later) using the equation of Weir (1949). Alcohol and caffeinated drinks were prohibited during the 24‐h measurement period, but water and non‐caffeinated herbal teas were available *ad libitum*.

Whilst residing in the respiration chamber, participants completed three bouts of 30 min step exercise (Reebok Aerobic Step ‐ height 150 mm; Reebok, Boston, Massachusetts, USA) at a step rate of 75 steps/min. Step exercise was performed at 0830 h (EX‐1), 1445 h (EX‐2), and 1915 h (EX‐3). The first step exercise bout (EX‐1) was performed fasted. Step rate was paced using a metronome and participants were visually monitored throughout. Step exercise (which incorporated ~6,750 steps; 75 steps/min × 90 min) was chosen to replicate the step count previously reported in free‐living older men (age: 70–74 years; 6,798 steps/d) (Lohne‐Seiler et al., 2014). Physical activity within the chamber (Activity_chamber_) was continuously measured by a radar transceiver working on the Doppler principle, and is expressed as the percent of time the participant was active (Ravussin et al., 1986).

### 2.8 EE and substrate oxidation via respiration chamber

Energy Expenditure and rates of carbohydrate and fat oxidation were calculated from continuous measurement of oxygen consumption (V̇O_2_) and carbon dioxide production (V̇CO_2_) by indirect calorimetry corrected for protein oxidation, using the equation of Brouwer (1957). Protein oxidation was determined from urinary nitrogen excretion measured over 12‐h periods (overnight: 2000–0800 h; daytime: 0800–2000 h) (Bingham et al., 1988; Brouwer, 1957). The RQ, which is an expression of relative fuel utilization, was calculated by dividing V̇CO_2_ by V̇O_2_. Carbohydrate, fat, and protein balances were determined as the difference between intake and oxidation.

Twenty‐four‐hour EE was partitioned into the following components. Total EE (TEE_chamber_) represented the EE between 2000 and 2000 h; SMR was determined as the lowest EE over a continuous 3 h period between 0000‐0600 h (Schoffelen & Westerterp, 2008); RMR was measured between 0700‐0800 h following awakening at 06:50 h with the participant supine, but awake. The first 20 and last 10 min were discarded, and RMR was calculated during the least restless consecutive 20 min period between 0720‐0750 h (Adriaens et al., 2003). The energy cost of step exercise was determined as the mean EE whilst participants were in steady‐state (McClave et al., 2003; Reeves et al., 2004). Other components of EE, including sedentary EE, AEE_chamber_, SPA, and DIT were calculated using the intercept method (see Figure S2) (Hall et al., 2016; Ravussin et al., 1986; Westerterp et al., 1999). Physical activity level (PAL_chamber_) was determined by dividing TEE_chamber_ by RMR. Components of EE and substrate oxidation are expressed as raw values and adjusted for body mass and composition (FFM and FM) using linear regression equations derived from the baseline chamber visit (Hall et al., 2016).

### 2.9 EE and physical activity via accelerometry

At baseline and week 12, participants wore a tri‐axial accelerometer (Actigraph GT9X; Actigraph, Pensacola, Florida, USA) continuously on the dominant wrist for 7 days in free‐living. At least 5 days of ≥10 h wear time was required for data to be included in the final analysis (Schrack et al., 2016). The accelerometer was sampled at 80 Hz and was analyzed in 60‐s epochs for total activity (Activity_accelerometry_; counts/d) using ActiLife (Version 6.13.4; Actigraph, Pensacola, Florida, USA). Activity_accelerometry_ was used to estimate AEE (AEE_accelerometry_) and PAL (PAL_accelerometry_) using the equations of Ekelund et al. (2004). Total EE (TEE_accelerometry_) was estimated by multiplying RMR measured inside the respiration chamber by PAL_accelerometry_. TEE_accelerometry_ and AEE_accelerometry_ are expressed as raw values and adjusted for body mass and composition using methods previously described.

### 2.10 Statistical analysis

Sample size was based on change (pre‐ vs. post‐intervention) and difference (between interventions post‐intervention) in RMR from previous respiration chamber studies in older adults (the former in older women) investigating the longitudinal effects of RE (Treuth et al., 1995) and a high protein diet (Drummen et al., 2020), respectively. Using G*Power (Version 3.1.9.2; Dusseldorf, Germany), a minimum of 34 participants (17/group) were required to observe a group‐by‐time interaction between RE and non‐exercise groups and PRO and CON groups for a mixed‐model ANCOVA with two covariates [*α* = 0.05; *β* = 0.8; effect size (Cohen's *f*) = 0.5].

Statistical analysis was performed using JASP Version 0.15 (https://jasp‐stats.org/). Data are presented as means ± SE. All data were checked for normality using the Shapiro‐Wilk test. Outliers (± >3SD from the group mean) were identified and removed. Non‐normally distributed data were transformed using appropriate transformation (i.e., log, square root, or reciprocal). Where transformation was unsuccessful, non‐parametric tests were utilized. Baseline characteristics were analyzed by independent samples *t*‐tests. A mixed‐model ANCOVA with time as the within‐subjects factor, group as the between‐subjects factor, and respective baseline values and dietary intervention group (PRO or CON, for RE vs. non‐exercise analyses) or RE participation (RE or non‐exercise, for PRO vs. CON analyses) included as covariates were performed on outcome variables. For exploratory sub‐analyses comparing the RE+CON and RE+PRO groups, outcomes were analyzed by a mixed‐model ANCOVA with baseline value included as a covariate only. Non‐normally distributed data were analyzed using the Scheirer‐Ray‐Hare two‐way ANOVA of ranks test. Longitudinal changes within groups were analyzed by 2‐tailed paired samples *t*‐tests. Correlations were analyzed using Pearson's partial correlation controlled for the intervention group. Significance was set at *p* < 0.05.

## 3 RESULTS

### 3.1 Participants

Thirty‐nine older men were randomized: Thirty three completed the study and 6 withdrew (see Figure S1 for participant flow). Baseline characteristics of the CON, PRO, non‐exercise, and RE groups are shown in Table 1 and characteristics of the RE+CON and RE+PRO groups are shown in Table S3.

**TABLE 1:** Baseline characteristics of participants^a^

	CON	PRO	*p* value^b^	Non‐exercise	RE	*p* value^b^
*n*	16	17	—	16	17	—
Age, years	67 ± 1	67 ± 1	0.74	66 ± 1	67 ± 1	0.48
Height, m	1.77 ± 0.01	1.76 ± 0.08	0.54	1.78 ± 0.01	1.75 ± 0.02	0.31
Body mass, kg	78.3 ± 2.5	80.3 ± 2.5	0.57	80.1 ± 2.2	78.6 ± 2.8	0.68
BMI, kg/m^2^	24.9 ± 0.6	25.9 ± 0.5	0.25	25.3 ± 0.6	25.5 ± 0.6	0.81
FFM, kg	59.0 ± 1.6	60.7 ± 1.7	0.47	60.6 ± 1.1	59.2 ± 2.0	0.55
SMM, kg	26.2 ± 0.7	27.2 ± 0.7	0.34	27.1 ± 0.5	26.3 ± 0.9	0.37
FM, kg	19.3 ± 1.4	19.6 ± 1.2	0.85	19.5 ± 1.5	19.4 ± 1.1	0.95
FM, %	24.3 ± 1.3	24.2 ± 1.0	0.96	23.9 ± 1.3	24.5 ± 1.0	0.75
TEE_chamber_, kcal/d	2439 ± 64	2490 ± 56	0.55	2473 ± 62	2457 ± 59	0.85
TEE_accelerometry_, kcal/d	2616 ± 81	2643 ± 74	0.81	2640 ± 81	2620 ± 75	0.86
RMR, kcal/day	1617 ± 50	1633 ± 46	0.81	1632 ± 50	1619 ± 46	0.86
PAL_chamber_	1.51 ± 0.02	1.53 ± 0.02	0.62	1.52 ± 0.02	1.53 ± 0.02	0.95
PAL_accelerometry_	1.56 ± 0.02	1.58 ± 0.02	0.60	1.56 ± 0.01	1.58 ± 0.02	0.26
Fasting plasma glucose, mmol/L	5.8 ± 0.2	5.9 ± 0.2	0.70	5.8 ± 0.2	5.8 ± 0.2	0.95
HOMA‐IR	2.5 ± 0.3	2.5 ± 0.3	0.98	2.7 ± 0.3	2.3 ± 0.3	0.39
Step count, steps/d	11,505 ± 666	11,840 ± 798	0.75	11,618 ± 775	11,733 ± 709	0.91
Activity_chamber_, %	16.9 ± 0.8	17.3 ± 1.1	0.81	16.9 ± 1.2	17.3 ± 0.7	0.74
Activity_accelerometry_, counts/day	276,039 ± 43,841	307,065 ± 39,979	0.61	257,482 ± 40,750	324,530 ± 41,496	0.26

Abbreviations: BMI, body mass index; FFM, fat‐free mass; FM, fat mass; HOMA‐IR, homeostatic model assessment of insulin resistance; PAL_accelerometry_, estimated physical activity level in free‐living by accelerometry; PAL_chamber_, physical activity level calculated inside the respiration chamber; RMR, resting metabolic rate; SMM, skeletal muscle mass; TEE_accelerometry_, estimated total energy expenditure in free‐living by accelerometry; TEE_chamber_, total energy expenditure calculated inside the respiration chamber.

^a^ Values are means ± SE.

^b^ *p* value refers to differences between groups analyzed by independent samples *t*‐test.

### 3.2 Exercise and supplement compliance

The mean attendance to the RE sessions was 98 ± 1% and did not differ between the RE+CON and RE+PRO groups (*p* = 0.98). All participants completed their prescribed repetitions for sets 1–2 of each exercise. During the final set (to volitional failure), the mean number of completed repetitions was 9.1 ± 0.2 and did not differ between the RE+CON and RE+PRO groups (*p* = 0.94). The mean compliance with the experimental supplements was 96 ± 1% and did not differ between CON and PRO groups (*p* = 0.27) or the RE+CON and RE+PRO groups (*p* = 0.97). Treatment allocation was unable to be judged by 81% of participants.

### 3.3 Dietary intake

Protein intake (g/d, g/kg/d, and % energy) increased in the PRO group greater than the CON group at weeks 6 and 12 (*p* < 0.001; Table 2). Similarly, protein intake (g/d, g/kg/d, and % energy) increased in the RE+PRO group greater than the RE+CON group at weeks 6 and 12 (*p* < 0.001; Table S4). Carbohydrate intake increased in the CON group greater than the PRO group at weeks 6 (g/d and % energy; *p* < 0.001) and 12 (g/d only; *p* < 0.001). In the RE+CON group, carbohydrate intake (g/d and % energy) increased greater than the RE+PRO group at weeks 6 and 12 (*p* < 0.001). Fat intake (g/d and % energy) and habitual total EI decreased in the PRO group greater than the CON group at week 12 (*p* < 0.05). No differences in any dietary marker occurred between the RE and non‐exercise groups.

**TABLE 2:** Self‐report dietary intake during the intervention period^a^

	Pooled	CON		PRO		*p* value^b^	Non‐exercise		RE		*p* value^b^
	Baseline	6 weeks	12 weeks	6 weeks	12 weeks		6 weeks	12 weeks	6 weeks	12 weeks	
Energy, kcal/d											
*Diet*	1999 ± 59	1898 ± 70	1971 ± 75	1967 ± 86	1862 ± 106* ^,^ ^c^	**0.01**	1850 ± 87	1831 ± 90	2013 ± 66	1993 ± 93	0.81
*Total*	1999 ± 59	2076 ± 75	2149 ± 77*	2158 ± 86*	2052 ± 106	**0.01**	2029 ± 91	2009 ± 90	2203 ± 66*	2183 ± 93	0.052
Protein, g/d											
*Diet*	82 ± 2	82 ± 4	80 ± 3	82 ± 4	80 ± 3	0.76	80 ± 3	78 ± 3	86 ± 4	82 ± 3	0.52
*Total*	82 ± 2	82 ± 4	80 ± 3	129 ± 3* ^,^ ^c^	126 ± 3* ^,^ ^c^	**< 0.001**	103 ± 7	101 ± 7	110 ± 7	106 ± 6	0.70
Protein, g/kg/d											
*Diet*	1.04 ± 0.03	1.04 ± 0.04	1.01 ± 0.04	1.05 ± 0.03	1.01 ± 0.03	0.81	0.99 ± 0.03	0.98 ± 0.03	1.10 ± 0.03	1.04 ± 0.04	0.31
*Total*	1.04 ± 0.03	1.04 ± 0.04	1.01 ± 0.04	1.61 ± 0.04* ^,^ ^c^	1.58 ± 0.04* ^,^ ^c^	**< 0.001**	1.27 ± 0.08	1.26 ± 0.09	1.39 ± 0.08	1.35 ± 0.08	0.07
Protein, %											
*Diet*	16.7 ± 0.4	17.3 ± 0.6	16.5 ± 0.7	17.1 ± 0.5	17.8 ± 0.8	0.17	17.3 ± 0.5	17.6 ± 0.9	17.1 ± 0.5	16.8 ± 0.7	0.96
*Total*	16.7 ± 0.4	15.8 ± 0.4	15.0 ± 0.6	24.3 ± 0.8* ^,^ ^c^	25.3 ± 1.1* ^,^ ^c^	**< 0.001**	20.4 ± 1.5	20.7 ± 1.8	19.9 ± 1.0	19.9 ± 1.3	0.99
Carbohydrate, g/d											
*Diet*	234 ± 8	211 ± 9	221 ± 11	210 ± 12	222 ± 12	0.99	197 ± 12	208 ± 12	223 ± 9	235 ± 10	0.56
*Total*	234 ± 8	255 ± 10	266 ± 11* ^,^ ^c^	210 ± 12	222 ± 12	**0.001**	218 ± 13	229 ± 13	245 ± 11	257 ± 11	0.59
Carbohydrate, %											
*Diet*	47.8 ± 1.5	44.9 ± 1.4	45.1 ± 1.7	42.8 ± 2.2	48.1 ± 1.4	0.12	43.1 ± 2.4	45.6 ± 1.6	44.6 ± 1.4	47.7 ± 1.6	0.85
*Total*	47.8 ± 1.5	49.3 ± 1.4^c^	49.7 ± 1.5^c^	38.9 ± 2.0	43.4 ± 1.3	**< 0.001**	43.2 ± 2.5	45.3 ± 1.5	44.7 ± 1.8	47.5 ± 1.7	0.81
Fat, g/d											
*Diet*	70 ± 3	70 ± 4	73 ± 5	75 ± 6	58 ± 6** ^,^ ^c^	**0.02**	71 ± 6	65 ± 5	74 ± 4	65 ± 6	0.72
*Total*	70 ± 3	70 ± 4	73 ± 5	76 ± 6	59 ± 6** ^,^ ^c^	**0.004**	72 ± 6	65 ± 5	74 ± 4	66 ± 5	0.89
Fat, %											
*Diet*	31.5 ± 1.0	33.0 ± 1.1	33.0 ± 1.5	34.1 ± 2.0	27.7 ± 1.4** ^,^ ^c^	**0.01**	34.3 ± 2.2	31.6 ± 1.6	32.9 ± 1.0	28.9 ± 1.5	0.50
*Total*	31.5 ± 1.0	30.2 ± 1.2	30.2 ± 1.4	31.4 ± 1.1	25.4 ± 1.4** ^,^ ^c^	**0.01**	31.5 ± 2.0	28.9 ± 1.5	30.2 ± 0.9	26.5 ± 1.4	0.55

Note

Diet = intake from habitual intake (excluding experimental supplements). Total = intake from habitual diet plus experimental supplements. Statistically significant values are indicated in bold.

^a^ Values are means ± SE. Baseline values for individual groups are not shown but no significant between‐group differences occurred.

^b^ *p* value refers to respective group‐by‐time interaction.

^c^ Significant difference between CON and PRO group at respective time point.

* *p* < 0.05 from baseline value

** *p* < 0.05 from week 6.

### 3.4 Body composition

**FIGURE 2:**
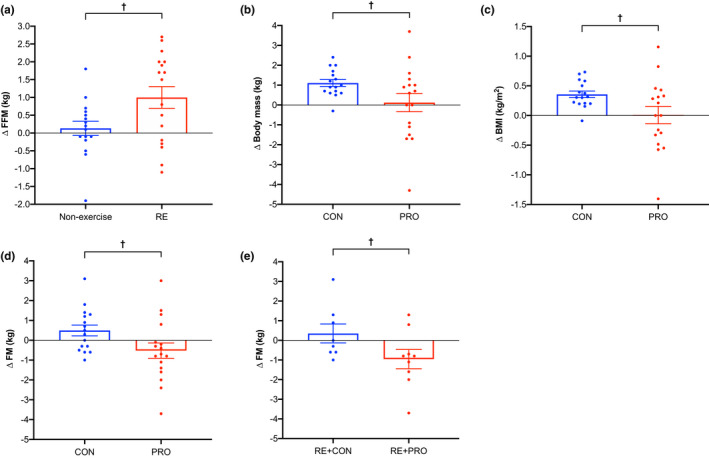
Changes in (a) FFM (kg) between RE (*n* = 17) and non‐exercise (*n* = 16) groups; (b) body mass (kg), (c) BMI (kg/m^2^), and (d) FM (kg) between PRO (*n* = 17) and CON (*n* = 16) groups; and (e) FM (kg) between RE+CON (*n* = 8) and RE+PRO (*n* = 9) groups over the intervention period (means ± SE). Circles represent individual data points. Data were analyzed using a mixed‐model ANCOVA with baseline value and dietary intervention (PRO or CON) included as covariates (panel a), baseline value and RE/non‐exercise included as covariates (panels b, c and d) and baseline value only included as a covariate (panel e). BMI, body mass index; FFM, fat‐free mass; FM, fat mass. ^†^ *p* < 0.05 between groups

Fat‐free mass significantly increased in the RE group greater than the non‐exercise group (*p* = 0.04; Figure 2a), but no differences occurred for any other body composition marker between these groups. Body mass (*p* = 0.04; Figure 2b) and BMI (*p *= 0.04; Figure 2c) significantly increased over time in the CON compared to the PRO group, and both absolute (*p* = 0.03; Figure 2d) and %FM (*p *= 0.04) decreased greater in the PRO group compared to CON group. No differences in skeletal muscle or FFM occurred between the PRO and CON groups (*p* ≥ 0.52). In the RE+CON group, body mass (*p* = 0.02) and BMI (*p *= 0.04) increased greater than the RE+PRO group. In contrast, absolute FM decreased greater in the RE+PRO group compared to the RE+CON group (*p* = 0.04; Figure 2e). No differences in skeletal muscle or FFM occurred between the RE+CON and RE+PRO groups (*p* ≥ 0.80).

### 3.5 EE and substrate oxidation

#### 3.5.1 TEE and energy balance

**FIGURE 3:**
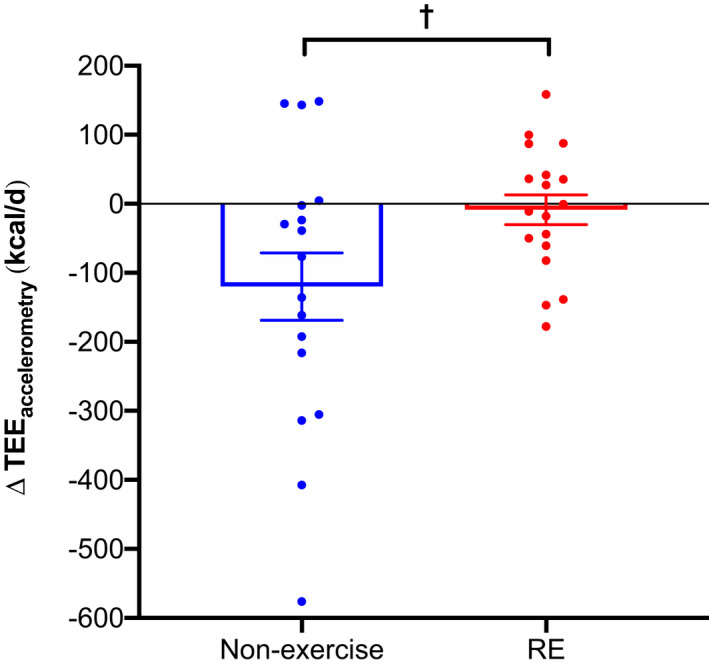
Change in unadjusted TEE_accelerometry_ (kcal/d) between RE (*n* = 17) and non‐exercise (*n* = 16) groups (means ± SE). Circles represent individual data points. Data were analyzed using a mixed‐model ANCOVA with baseline value and dietary intervention (PRO or CON) included as covariates. ^†^ *p* < 0.05 between groups. TEE_accelerometry_, total energy expenditure estimated by accelerometry

TEE_accelerometry_ significantly decreased over time in the non‐exercise group greater than the RE group (*p* = 0.03; Figure 3 and Table 3), which remained significant when adjusted for body mass and composition (*p *< 0.05). No differences in TEE_accelerometry_ occurred over time between the CON and PRO groups (*p* = 0.55) or the RE+CON and RE+PRO groups (*p* = 0.80; Table S5). No between‐group differences occurred for unadjusted or adjusted TEE_chamber_.

**TABLE 3:** Energy expenditure and 24‐h substrate oxidation and balances for each treatment group at baseline and 12 weeks^a^

	CON		PRO		*p* value^b^	Non‐exercise		RE		*p* value^b^
	Baseline	12 weeks	Baseline	12 weeks		Baseline	12 weeks	Baseline	12 weeks	
TEE_chamber_, kcal/d	2439 ± 64	2392 ± 65^#^	2490 ± 56	2445 ± 60^#^	0.99	2473 ± 62	2412 ± 62^#^	2457 ± 58	2427 ± 63	0.18
TEE_accelerometry_, kcal/d	2616 ± 81	2570 ± 72	2643 ± 73	2558 ± 67	0.55	2640 ± 81	2513 ± 66^#^	2621 ± 75	2612 ± 70	**0.03**
Sedentary EE, kcal/d	1736 ± 45	1729 ± 53	1723 ± 52	1763 ± 49	0.28	1744 ± 54	1716 ± 44	1716 ± 44	1775 ± 48	**0.049**
RMR, kcal/d	1617 ± 50	1628 ± 46	1633 ± 46	1621 ± 42	0.49	1632 ± 50	1592 ± 42	1619 ± 46	1656 ± 44	**0.03**
SMR,^c^ kcal/dx	1562 ± 52	1581 ± 50	1599 ± 42	1621 ± 42	0.84	1582 ± 44	1577 ± 45	1582 ± 49	1628 ± 47^#^	**< 0.001**
AEE_chamber_, kcal/d	702 ± 36	663 ± 26	767 ± 32	682 ± 30	0.60	729 ± 33	695 ± 31	741 ± 36	652 ± 26^#^	0.11
AEE_accelerometry_, kcal/d	772 ± 46	750 ± 40	805 ± 42	779 ± 50	0.82	752 ± 43	799 ± 56	823 ± 44	733 ± 33	**0.049**
SPA, kcal/d	390 ± 27	346 ± 20	433 ± 31	338 ± 26	0.36	410 ± 28	365 ± 25	414 ± 31	320 ± 19^#^	0.09
DIT, kcal/d	148 ± 18	152 ± 17	124 ± 29	142 ± 25	0.93	134 ± 25	131 ± 26	137 ± 24	162 ± 16	0.30
DIT, % of EI	6.4 ± 0.8	6.4 ± 0.7	4.9 ± 1.2	5.8 ± 1.1	0.96	5.5 ± 1.0	5.4 ± 1.1	5.8 ± 1.0	6.8 ± 0.7	0.31
PAL_chamber_	1.51 ± 0.02	1.47 ± 0.02	1.53 ± 0.02	1.51 ± 0.02	0.24	1.52 ± 0.02	1.52 ± 0.02	1.53 ± 0.02	1.47 ± 0.03^#^	0.06
PAL_accelerometry_	1.56 ± 0.02	1.55 ± 0.02	1.58 ± 0.02	1.57 ± 0.02	0.82	1.56 ± 0.02	1.57 ± 0.02	1.58 ± 0.02	1.55 ± 0.01	**0.049**
Protein oxidation, g/d	85 ± 5	80 ± 4	86 ± 4	105 ± 5^#^	**< 0.001**	87 ± 4	94 ± 6	84 ± 4	92 ± 5	0.99
Carbohydrate oxidation, g/d	254 ± 10	248 ± 9	248 ± 11	242 ± 11	0.82	256 ± 11	247 ± 13	247 ± 10	243 ± 7	0.90
Fat oxidation, g/d	101 ± 6	101 ± 6	109 ± 3	98 ± 4	0.14	103 ± 6	98 ± 5	107 ± 4	101 ± 4	0.86
RQ	0.85 ± 0.01	0.84 ± 0.01	0.84 ± 0.01	0.84 ± 0.01	0.99	0.85 ± 0.01	0.85 ± 0.01	0.84 ± 0.01	0.84 ± 0.01	0.84
Protein balance, g/d	29 ± 5	30 ± 3	27 ± 4	7 ± 5^#^	**< 0.001**	27 ± 5	15 ± 5^#^	29 ± 4	22 ± 4	0.38
Carbohydrate balance, g/d	47 ± 9	41 ± 9	46 ± 8	55 ± 9	0.26	45 ± 9	45 ± 10	48 ± 8	52 ± 7	0.62
Fat balance, g/d	−7 ± 5	−7 ± 5	−12 ± 5	−1 ± 5^#^	0.09	−7 ± 4	−4 ± 5	−12 ± 6	−5 ± 4	0.82
EB_chamber_, kcal/d	28 ± 47	52 ± 38	14 ± 48	49 ± 34	0.85	34 ± 52	33 ± 40	8 ± 43	67 ± 32	0.27
EB_free‐living_, kcal/d	–637 ± 68	−421 ± 59^#^	−626 ± 72	−506 ± 80^#^	0.16	−723 ± 66	−504 ± 67^#^	−545 ± 67	−428 ± 74^#^	0.13

Abbreviations: AEE_accelerometry_, estimated activity energy expenditure by accelerometry; AEE_chamber_, activity energy expenditure calculated inside the respiration chamber; DIT, diet‐induced thermogenesis; EB_chamber_, calculated energy balance inside the respiration chamber; EB_free‐living_, estimated energy balance in free‐living; PAL_accelerometry_, estimated physical activity level by accelerometry; PAL_chamber_, physical activity level calculated inside the respiration chamber; RMR, resting metabolic rate; RQ, respiratory quotient; SMR, sleeping metabolic rate; SPA, spontaneous physical activity; TEE_accelerometry_, estimated total energy expenditure by accelerometry; TEE_chamber_, total energy expenditure calculated inside the respiration chamber. ^#^ *p* < 0.05 from baseline value.

^a^ Values are means ± SE. Energy expenditure and substrate oxidation values are reported unadjusted.

^b^ *p* value refers to respective group‐by‐time interaction.

^c^ CON, *n* = 15; Exercise, *n* = 16 (*n* = 1 outlier >3SD from mean removed from each group as described in the main text).

Across the whole sample, energy balance inside the respiration chamber (EB_chamber_) was 20 ± 33 and 50 ± 25 kcal/d at baseline and 12 weeks, respectively (Table 3). No between‐group differences occurred (*p* ≥ 0.27). EB_chamber_ at baseline and 12 weeks was checked by comparing obtained energy balance by a fictive energy balance of zero. No groups’ EB_chamber_ significantly differed from zero at either baseline or 12 weeks. Energy balance in free‐living (EB_free‐living_) was –631 ± 49 and –465 ± 50 kcal/d at baseline and 12 weeks, respectively, across the whole sample. No between‐group differences occurred (*p *≥ 0.13). Negative EB_free‐living_ in all groups was confirmed using the methodology previously described. As participants in the CON and RE+CON groups gained body mass and no changes were observed in the PRO or RE+PRO groups, underreporting of self‐report EI (~20%) largely was assumed to explain the observed negative EB_free‐living_.

#### 3.5.2 24‐h substrate oxidation and balance

**FIGURE 4:**
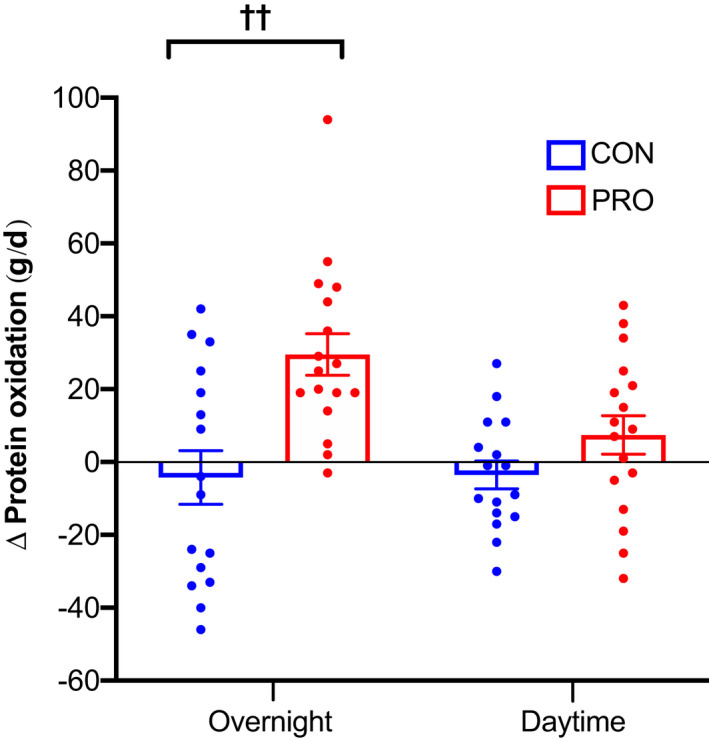
Changes in unadjusted overnight and daytime protein oxidation (g/d) between PRO (*n* = 17) and CON (*n *= 16) groups over the intervention period (means ± SE). Circles represent individual data points. Data were analyzed using a mixed‐model ANCOVA with baseline value and RE/non‐exercise included as covariates. ^††^ *p* < 0.01 between groups

Twenty‐four‐hour protein oxidation increased over time in the PRO group greater than the CON group (*p* < 0.001; Table 3), which remained significant when adjusted for body mass and composition (*p* < 0.001). Similarly, 24‐h protein oxidation significantly increased over time in the RE+PRO group greater than the RE+CON group (*p* < 0.001; Table S5). The increase in 24‐h protein oxidation following the high protein diet was driven by a rise in overnight protein oxidation, which increased by 30 ± 6 g/d (*p* < 0.001) (Figure 4). No differences in 24‐h (*p* = 0.99) or overnight protein oxidation (*p* = 0.94) occurred between the RE and non‐exercise groups and no between‐group differences occurred for daytime protein oxidation (*p* ≥ 0.19; see Figure 4 for PRO vs. CON). Protein balance was positive in all groups at baseline but decreased in the PRO group compared to the CON group when protein intake was returned to the chamber diet at week 12 (*p* < 0.001). Likewise, protein balance significantly decreased over time in the RE+PRO compared to the RE+CON group (*p* = 0.01). Protein balance did not differ over time between the RE and non‐exercise groups (*p* = 0.38). No between‐group differences in 24‐h oxidation or balance of carbohydrate or fat, or RQ, were observed over the course of the study.

#### 3.5.3 Resting, sedentary, and sleeping EE and substrate oxidation

**FIGURE 5:**
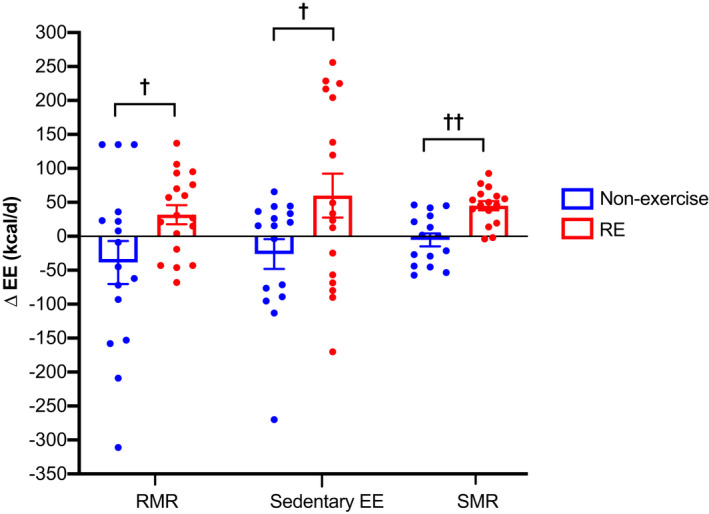
Changes in unadjusted resting metabolic rate (RMR), sedentary EE and sleeping metabolic rate (SMR) (kcal/d) between RE (*n* = 17 for RMR and sedentary EE; *n* = 16 for SMR) and non‐exercise (*n* = 16 for RMR and sedentary EE; *n *= 15 for SMR) groups over the intervention period (means ± SE). Two participants SMR data (*n* = 1 participant from the RE and non‐exercise groups) were removed from analysis as described in the main text. Circles represent individual data points. Data were analyzed using a mixed‐model ANCOVA with baseline value and dietary intervention (PRO or CON) included as covariates. EE, energy expenditure. ^†^ *p* < 0.05 between groups. ^††^ *p* < 0.01 between groups

Resting metabolic rate (*p* = 0.03), sedentary EE (*p* = 0.049), and SMR (*p *< 0.001) significantly increased in the RE group greater than the non‐exercise group (Table 3 and Figure 5). When adjusted for measured changes in body composition, RMR (*p* = 0.007) and SMR (*p* = 0.008) remained significantly increased, but sedentary EE did not (*p* = 0.35). No differences in RMR, sedentary EE or SMR occurred between the PRO and CON groups (*p* ≥ 0.28) or the RE+CON and RE+PRO groups (*p* ≥ 0.55; Table S5). Two participants’ SMR data [*n* = 1 participant from both the CON and RE groups (the latter of which *n* = 1 participant from the RE+CON group)] were removed from analysis due to poor sleep quality at baseline, which led to elevated baseline SMR and the resulting ΔSMR being clear outliers (>3SD from group mean).

**FIGURE 6:**
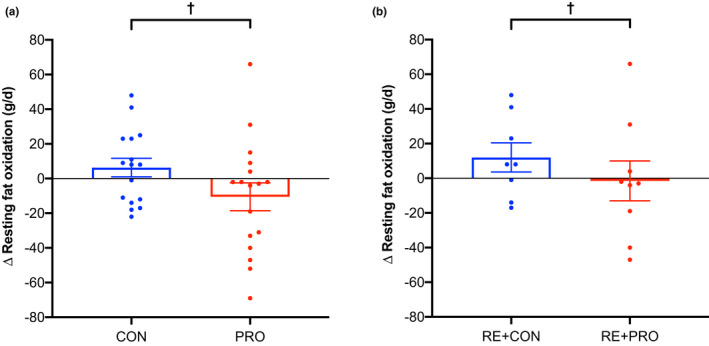
Change in unadjusted resting fat oxidation (g/d) between (a) PRO (*n* = 17) and CON (*n *= 16) groups and (b) RE+CON (*n* = 8) and RE+PRO (*n* = 9) groups over the intervention period (means ± SE). Circles represent individual data points. Data were analyzed using a mixed‐model ANCOVA with baseline value and RE/non‐exercise included as covariates (panel A) and baseline value only included as a covariate (panel B). ^†^ *p* < 0.05 between groups

Resting fat oxidation significantly decreased in the PRO group compared to the CON group (*p* = 0.01; Figure 6a), which remained significant when adjusted for body mass (*p *= 0.008) and composition (*p* = 0.01). In the RE+CON group, resting fat oxidation significantly increased compared to the RE+PRO group (*p* = 0.01; Figure 6b) and remained significant when adjusted for body mass (*p *= 0.004) and composition (*p* = 0.01). No between‐differences occurred for resting or sleeping carbohydrate oxidation, RQ, or sleeping fat oxidation.

#### 3.5.4 Activity EE

**FIGURE 7:**
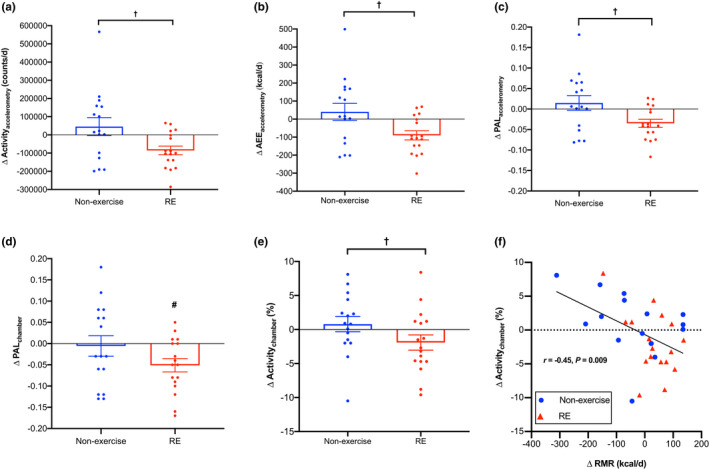
Changes in (a) Activity_accelerometry_ (counts/d); (b) unadjusted AEE_accelerometry_ (kcal/d); (c) PAL_accelerometry_; (d) PAL_chamber;_ and (e) Activity_chamber_ (%) between RE (*n* = 17) and non‐exercise (*n* = 16) groups (means ± SE). Circles represent individual data points. Panel F shows the correlation between ΔRMR (kcal/d) and Δactivity_chamber_ (%). Circles and triangle symbols in panel (f) represent individual data points in the non‐exercise and RE groups, respectively. Data were analyzed using a mixed‐model ANCOVA with baseline value and dietary intervention (PRO or CON) included as covariates (panels a to e) and by Pearson's partial correlation (controlled for intervention group; panel f). Activity_accelerometry_, physical activity measured by accelerometry; Activity_chamber_, physical activity measured inside the respiration chamber; AEE_accelerometry_, activity energy expenditure estimated in free‐living by accelerometry; PAL_accelerometry_, estimated physical activity level by accelerometry; PAL_chamber_, physical activity level calculated inside the respiration chamber; RMR, resting metabolic rate; ^†^ *p* < 0.05 between groups. ^#^ *p* < 0.05 from baseline

Activity_accelerometry_ (*p* = 0.046; Figure 7a) and AEE_accelerometry_ (*p* = 0.049; Figure 7b) significantly decreased in the RE group compared to non‐exercise group, the latter of which remained significant when adjusted for body mass (*p* = 0.048) and composition (*p* = 0.046). No differences occurred between the PRO and CON groups (*p* ≥ 0.82; Table 3) or the RE+CON and RE+PRO groups (*p* ≥ 0.10). AEE_chamber_ did not differ over time between groups (*p* ≥ 0.11); however, significant within‐group decreases were observed in the RE (*p* = 0.001) and RE+PRO groups (*p* = 0.03; Table S5), which remained significant when adjusted for body mass and composition (*p* < 0.05). The decrease in AEE_accelerometry_ and AEE_chamber_ following RE led to a greater decrease in PAL_accelerometry_ (*p* = 0.049; Figure 7c) and PAL_chamber_ (*p* = 0.06; Figure 7d) compared to the non‐exercise group. The decrease in AEE_chamber_ following RE was driven by a decrease in SPA (*p* < 0.001), which tended to decrease greater than the non‐exercise group (*p* = 0.09). Decreased activity_chamber_ (*p* = 0.049, Figure 7e) as opposed to SPA per % of activity (*p* = 0.35) caused the decrease in SPA in the RE compared to the non‐exercise group. The Δactivity_chamber_ significantly inversely correlated with ΔRMR (*r *= −0.45, *p *= 0.009; Figure 7f). No differences occurred between the RE+CON and RE+PRO groups.

#### 3.5.5 Step exercise EE and substrate oxidation

**FIGURE 8:**
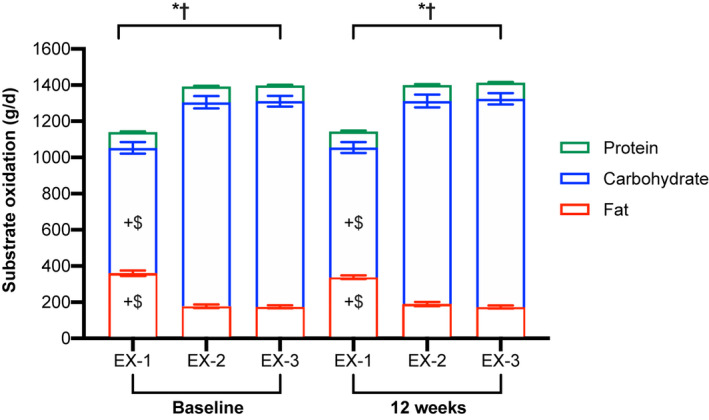
Carbohydrate, fat and protein oxidation (g/d; unadjusted) during the three step exercise bouts performed at 75 steps/min at baseline and 12 weeks (*n *= 33; means ± SE). EX‐1, step exercise bout 1 (0830 h in the fasted state); EX‐2, step exercise bout 2 (1445 h); EX‐3, step exercise bout 3 (1915 h). Baseline and week 12 data were analyzed by repeated measures ANOVA. *Significant main effect of exercise bout for fat oxidation. ^†^Significant main effect of exercise bout for carbohydrate oxidation. ^+^ *p* < 0.001 compared to EX‐2. ^$^ *p *< 0.001 compared to EX‐3

At baseline and 12 weeks, across the whole sample, fat oxidation was significantly greater and carbohydrate oxidation was significantly less during the fasted step exercise bout (EX‐1) than during both EX‐2 (*p* < 0.001) and EX‐3 (*p* < 0.001; Figure 8). Energy expenditure did not differ between step exercise bouts at baseline (*p* ≥ 0.10). In contrast, at 12 weeks, EE during EX‐1 (4.7 ± 0.1 kcal/min) was significantly less than during both EX‐2 (4.9 ± 0.1 kcal/min; *p* < 0.001) and EX‐3 (4.8 ± 0.1 kcal/min; *p* < 0.001). Following the intervention, no significant between‐group differences in unadjusted or adjusted EE or substrate oxidation occurred overtime for any step exercise bout (see Table 4 and Table S6 for unadjusted data).

**TABLE 4:** Step exercise energy expenditure and substrate oxidation for each treatment group at baseline and 12 weeks^a^.

	CON		PRO		*p* value^b^	Non‐exercise		RE		*p* value^b^
	Baseline	12 weeks	Baseline	12 weeks		Baseline	12 weeks	Baseline	12 weeks	
**EX−1**										
EE, kcal/min	4.6 ± 0.2	4.6 ± 0.2	4.8 ± 0.2	4.7 ± 0.2	0.56	4.8 ± 0.2	4.6 ± 0.2	4.7 ± 0.1	4.7 ± 0.2	0.33
Carbohydrate oxidation, g/d	671 ± 43	696 ± 34	713 ± 47	738 ± 51	0.76	655 ± 50	700 ± 51	729 ± 40	734 ± 36	0.80
Fat oxidation, g/d	349 ± 22	339 ± 16	369 ± 20	335 ± 17	0.37	382 ± 23	340 ± 16	339 ± 17	334 ± 17	0.30
Protein oxidation, g/d	87 ± 6	84 ± 5	88 ± 4	96 ± 5	0.28	89 ± 4	92 ± 5	87 ± 5	86 ± 6	0.83
RQ	0.84 ± 0.01	0.84 ± 0.01	0.84 ± 0.01	0.85 ± 0.01	0.61	0.83 ± 0.01	0.84 ± 0.01	0.85 ± 0.01	0.85 ± 0.01	0.71
**EX−2**										
EE, kcal/min	4.7 ± 0.2	4.7 ± 0.2	4.9 ± 0.1	5.0 ± 0.2	0.96	4.8 ± 0.2	4.9 ± 0.2	4.9 ± 0.1	4.9 ± 0.1	0.40
Carbohydrate oxidation, g/d	1095 ± 53	1073 ± 47	1160 ± 43	1165 ± 50	0.37	1106 ± 50	1109 ± 58	1149 ± 47	1131 ± 42	0.75
Fat oxidation, g/d	174 ± 19	197 ± 14	179 ± 11	185 ± 16	0.55	180 ± 19	191 ± 16	174 ± 11	190 ± 15	0.95
Protein oxidation, g/d	87 ± 6	84 ± 5	88 ± 4	96 ± 5	0.28	89 ± 4	92 ± 5	87 ± 5	86 ± 6	0.83
RQ	0.91 ± 0.01	0.91 ± 0.01	0.91 ± 0.01	0.91 ± 0.01	0.54	0.91 ± 0.01	0.91 ± 0.01	0.91 ± 0.01	0.91 ± 0.01	0.49
**EX−3**										
EE, kcal/min	4.7 ± 0.2	4.7 ± 0.2	4.9 ± 0.1	5.0 ± 0.2	0.37	4.8 ± 0.2	4.8 ± 0.2	4.9 ± 0.1	4.9 ± 0.2	0.82
Carbohydrate oxidation, g/d	1104 ± 45	1110 ± 41	1168 ± 42	1191 ± 46	0.46	1120 ± 45	1113 ± 46	1153 ± 42	1189 ± 42	0.25
Fat oxidation, g/d	171 ± 16	173 ± 15	177 ± 10	173 ± 10	0.83	174 ± 13	182 ± 13	174 ± 12	165 ± 11	0.21
Protein oxidation, g/d	87 ± 6	84 ± 5	88 ± 4	96 ± 5	0.28	89 ± 4	92 ± 5	87 ± 5	86 ± 6	0.83
RQ	0.92 ± 0.01	0.92 ± 0.01	0.91 ± 0.01	0.92 ± 0.01	0.51	0.91 ± 0.01	0.91 ± 0.01	0.91 ± 0.01	0.92 ± 0.01	0.06

Note

Energy expenditure and substrate oxidation values are reported unadjusted. All step exercise bouts were performed at a step rate of 75 steps/min for 30 min. Protein oxidation data has been added for completeness but was obtained from one urine sample between 0800 and 2000 h.

Abbreviations: EE, energy expenditure; EX‐1, step exercise bout 1 (0830 h in the fasted state); EX‐2, step exercise bout 2 (1445 h); EX‐3, step exercise bout 3 (1915 h); RQ, respiratory quotient.

^a^ Values are means ± SE.

^b^ *p* value refers to respective group‐by‐time interaction.

* *p* < 0.05 from baseline value.

#### 3.5.6 Diet‐induced thermogenesis

Diet‐induced thermogenesis was similar between groups at baseline (*p *≥ 0.31; Table 3 and Table S5). A high inter‐individual variability was observed (range −3.1–12.5%). At 12 weeks when protein intake was returned to the baseline chamber diet, no within‐ or between‐group differences occurred.

## 4 DISCUSSION

The present study is the first to examine changes in 24‐h EE, substrate oxidation, and body composition in older adults (60–75 years) following RE and a high protein diet via whey protein supplementation [which aimed to increase dietary protein intake by ≥0.4 g/kg/d to 1.6 g/kg/d (~25% of EI)]. The main findings were: (i) RE significantly increased FFM, RMR, SMR and sedentary EE and mitigated a decline in free‐living TEE compared to non‐exercise; however, decreased AEE and PAL; (ii) a high protein diet (~1.6 g/kg/day and ~25% of EI) via whey protein supplementation improved body weight maintenance and reduced FM compared to control, but reduced resting fat oxidation and increased overnight protein oxidation, which subsequently decreased 24‐h protein balance; and (iii) a high protein diet via whey protein supplementation combined with RE synergistically improved body weight maintenance and reduced FM compared to RE and a carbohydrate control, but did not significantly augment increases in FFM or EE components.

Twelve weeks of RE resulted in increased RMR, sedentary EE, and SMR. These increases were likely largely a result of the energetic cost of increased FFM (Hunter et al., 2004). The rise in SMR observed in the present study is in line with those reported in older women (67 ± 1 years) (Treuth et al., 1995); however, the increase in RMR is considerably less than studies that reported increases of ~7–9% (Campbell et al., 1994; Hunter et al., 2000; Pratley et al., 1994; Treuth et al., 1995 ). Fat‐free mass increased by a similar or greater magnitude in the present study than that reported by the majority of these studies (Campbell et al., 1994; Pratley et al., 1994; Treuth et al., 1995); therefore, inconsistencies may be due to alternative factors, including participant training status, sex, changes in rates of MPS and sympathetic nervous system activity, and differences in the timing of post‐intervention RMR measurement relative to termination of the final RE session (Geisler et al., 2016b; Geisler & Müller, 2017; Schutz, 2011; Speakman & Selman, 2003).

The present study reports that a high protein diet (~1.6 g/kg/d and ~25% of EI) via whey protein supplementation aided maintenance of body mass and reductions in both absolute and %FM compared to an isocaloric carbohydrate control. Similarly, a high protein diet combined with RE also aided body weight maintenance and reduced FM compared to RE combined with control. These findings coincide with studies that demonstrated that a high protein diet effectively maintains energy balance and body mass and decreases FM (Clifton et al., 2014; Drummen et al., 2018; Kim et al., 2016; Martens et al., 2015), and augments RE‐induced FM reduction (Bell et al., 2017; Liao et al., 2017). As no effect of a high protein diet on EE components was observed, compensatory reductions in habitual fat and EI might partially explain the present findings. Though, in agreement with many previous studies as reviewed by Ravelli and Schoeller (2020), it must be emphasized that significant underreporting (~20%) of self‐report EI was observed in this study compared to that of estimated free‐living TEE by accelerometry. Consequently, self‐report dietary intake data in this study should be interpreted cautiously.

In the present study, we anticipated that EE components would increase following a high protein diet, mainly due to the energetic cost of increased FFM (Drummen et al., 2018). However, no individual or augmented increases in EE were observed, which contradicts studies in younger adults that demonstrated protein‐induced increases in SMR (Bray et al., 2015; Martens et al., 2015), RMR (Bray et al., 2012), and TEE (Bray et al., 2012, 2015). The findings of this study also contradict those of Drummen et al. (2020), who reported an increase in RMR following ~34 months of a high compared to a moderate protein diet in older adults. Though, it must be noted that Drummen et al. (2020) only measured EE components post‐intervention, so the pre‐post energetic effects of the intervention used in this study are unknown. Contrary to these studies, however, the findings of the present study are in agreement with studies that demonstrated no synergistic effects of combined RE and increased dietary protein intake on RMR (Amamou et al., 2017; Campbell et al., 1994; Maltais et al., 2016; Weinheimer et al., 2012) and no effect of increased dietary protein intake alone on either resting (Luger et al., 2013; Negro et al., 2019) or SMR in older adults (Drummen et al., 2020).

The most likely explanation for the present and above‐cited null findings is due to a lack of protein‐induced increase in FFM (Amamou et al., 2017; Campbell et al., 1994; Maltais et al., 2016; Weinheimer et al., 2012). These findings are consistent with others that observed no individual (Björkman et al., 2020; Kim et al., 2012; Kirk et al., 2020; Verreijen et al., 2017; Zhu et al., 2015) or synergistic effects (Arnarson et al., 2013; Candow et al., 2006; Dulac et al., 2021; Holm et al., 2008; Holwerda et al., 2018; Kirk et al., 2020; Kukuljan et al., 2009; Leenders et al., 2013; Thomson et al., 2016; Verdijk et al., 2009) of increased dietary protein intake on FFM in healthy older adults habitually consuming ample amounts of dietary protein (~1.0–1.2 g/kg/d). In contrast, studies conducted in older adults who were either sarcopenic/frail or reported lower habitual intakes of dietary protein (<1.0 g/kg/d) have observed both individual (Bauer et al., 2015; Bo et al., 2019; Kang et al., 2020; Park et al., 2018; ten Haaf et al., 2019) and augmented increases in FFM (Kang et al., 2019; Rondanelli et al., 2016, 2018; Tieland et al., 2012b; Yamada et al., 2019; Zdzieblik et al., 2015). The relatively good health status of participants who habitually consumed adequate amounts of dietary protein in this study may have masked any effects of increased intake via supplementation.

It must also be noted that in the present study, participants in the high protein diet groups were not fed the same diet whilst residing in the respiration chamber post‐intervention, which contrasts with previous respiration chamber studies that demonstrated longitudinal protein‐induced increases in components of EE (Bray et al., 2015; Martens et al., 2015). As the acute energetic effects of a high protein diet are well‐known (Drummen et al., 2018), these methodological differences may also explain the contrasting findings between the present and previous studies. However, as previously detailed in the methods, we opted for this methodology to determine the effects on FFM and subsequent effects on energetics in the absence of acute protein‐induced effects on EE. Nevertheless, the present study still observed increases in overnight protein oxidation and we have previously reported a 73% increase in awakening cortisol concentration following the high protein diet in this cohort (Griffen et al., 2022), suggesting an increase in gluconeogenesis. These increases did not translate into increased EE however as per previous studies (Drummen et al., 2018; Veldhorst et al., 2009), largely due to the increase in protein oxidation being spared for reduced fat oxidation. This finding is consistent with a previous study on younger adults (Bray et al., 2015).

While RE increased several EE components, TEE, measured both inside the respiration chamber and estimated in free‐living by accelerometry, did not increase. Although, it should be noted that RE did mitigate a decrease in free‐living TEE compared to non‐exercise. The lack of increase in TEE in free‐living and whilst residing in the respiration chamber following RE was caused by a decrease in PAL and AEE, an observation frequently seen in older adults following exercise interventions (Westerterp, 2018c). The decrease in AEE was due to a decrease in SPA. In fact, inside the respiration chamber, non‐exercise activity decreased by 1.9 ± 1.1% following RE, which equated to a 27 min (1440 min × 0.019) reduction. These findings are consistent with others (Hunter et al., 2018; Melanson, 2017) and have been suggested to occur due to increased training frequency‐induced fatigue (Hunter et al., 2018) and/or energy compensation to maintain energy balance (Careau et al., 2021; Hall et al., 2012). In support of the latter, we report an inverse correlation between change in RMR and change in non‐exercise physical activity whilst participants resided in the respiration chamber. As prior work has shown that increasing AEE may be an effective strategy in the defense against adiposity (Kotz et al., 2017), these findings somewhat question the use of RE in older adults to increase habitual physical activity and offset sarcopenic obesity. However, as the present study was of relatively short duration, it may be that older individuals require a longer duration than 12 weeks to adapt to frequent RE training. Therefore, future work should investigate AEE following a longer period of RE training in this population.

The increase in 24‐h protein oxidation following 12 weeks of a high protein diet via whey protein supplementation concurs with studies in both young (Bray et al., 2015; Martens et al., 2015; Pannemans et al., 1995a; Robinson et al., 1990) and older adults (Drummen et al., 2020; Pannemans et al., 1995b, 1998). In the present study, raised overnight as opposed to daytime protein oxidation stimulated the 24‐h increase, consistent with prior work (Price et al., 1994). Furthermore, the present study also established when dietary protein intake was returned to that of the chamber diet, 24‐h protein balance was significantly reduced. This outcome is in agreement with others that established a lower net protein balance in older men following habituation to a high protein diet (Højfeldt et al., 2020) and a reduced nitrogen balance following termination of a high protein diet until nitrogen output matched the new level of intake (Waterlow, 1999). Previously in this cohort, as formerly mentioned, we observed an increase in awakening salivary cortisol in addition to increased fasting plasma myostatin concentration following termination of the high protein diet used in this study (Griffen et al., 2022). Together with the increase in overnight protein oxidation observed, this indicates a decrease in protein breakdown whilst fasting. These findings indicate a potential drawback of older adults habitually consuming high intakes (~1.6 g/kg/d) of dietary protein and suggest that older individuals should refrain from drastically reducing protein intake once commenced on a high protein diet to minimize transient periods of reduced protein balance.

During the step exercise bouts, greater rates of fat oxidation were observed in the fasted step exercise bout compared to step exercise performed postprandially at 1445 and 1915 h at both baseline and 12 weeks. These findings are similar to that of Edinburgh et al. (2020), who observed higher rates of fat oxidation following an acute bout of fasted exercise compared to exercise performed following consumption of breakfast. Edinburgh et al. (2020) also observed a greater sustained increase in fat oxidation when exercise was performed in the fasted state for 6 weeks alongside improvements in insulin sensitivity. Based on these data, research quantifying the longitudinal metabolic response of fasted exercise in the elderly, of whom are at greater risk of metabolic dysfunction (Hunter et al., 2019), is warranted to determine the safety and effectiveness of mitigating age‐related metabolic disease.

To date, only a few studies of ≥4 weeks in duration have assessed the effects of either a dietary or exercise intervention on multiple components of EE and substrate oxidation using respiration chambers in older adults (Bush et al., 2018; Drummen et al., 2020; Morio et al., 1998; Treuth et al., 1995). The investigation of both the individual and combined effects of a dietary and exercise intervention over 12 weeks on these outcomes measured inside a respiration chamber, which was supplemented with an estimation of EE in free‐living by accelerometry, are key strengths and novel aspects of this study. A limitation of this study is the use of pooled data for the main analysis, which may have impacted the individual effects of RE and a high protein diet via whey protein supplementation. A second limitation is the relatively small number of participants in our exploratory sub‐analysis investigating the synergistic effects of RE and a high protein diet. The sample sizes of these groups where, however, congruent with previous respiration chamber studies (Apolzan et al. 2014; Bray et al., 2015; Melanson et al., 2007; Westerterp‐Plantenga et al., 2009). Other limitations include the failure to check protein intake during the intervention period by measured urinary nitrogen, omission of hydration status assessment prior to BIA measurement, and inclusion of only men who were healthy and had a normal‐to‐overweight BMI. Women were excluded based on age‐related sex differences in the decline in RMR (Geisler et al., 2016b). Individuals with obesity were excluded due to differences in metabolic profile (Perna et al., 2017) and due to the preventative nature of this study. Investigation of these groups and older adults with insufficient habitual protein intakes (<1 g/kg/d) should form future work.

In conclusion, 12 weeks of RE significantly increased FFM, RMR, SMR, and sedentary EE compared to non‐exercise. Conversely, RE decreased AEE and PAL. A high protein diet (~1.6 g/kg/d and ~25% of EI) via whey protein supplementation aided body weight maintenance and reduced FM compared to control. However, decreased resting fat oxidation and increased overnight protein oxidation, which subsequently reduced 24‐h protein balance. Resistance exercise combined with a high protein diet via whey protein supplementation synergistically improved body weight maintenance and reduced FM compared to RE and control but did not significantly augment increases in FFM or EE components.

## 5 COMPETING INTERESTS

The whey protein supplement used in this study (Instantized BiPRO) was supplied by Agropur, Quebec, Canada. Agropur provided the supplement free of charge but had no involvement in data collection or analysis of this study. The authors declare no other conflicts of interest.

## AUTHORS CONTRIBUTIONS

Corbin Griffen designed the study, conducted and analyzed the data, and wrote the manuscript; John Hattersley provided support in the design, conduct and analysis of the study and contributed to writing and critical review of the manuscript; Derek Renshaw provided support in the design of the study, and contributed to writing and critical review of the manuscript; Michael Duncan provided support in the design of the study, and contributed to writing and critical review of the manuscript; Martin O. Weickert served as primary clinical advisor and critically reviewed the manuscript. All authors have read and approved the final version of the manuscript and agree with the order of author presentation.

## ENDNOTE

At the request of the author(s), readers herein are alerted to the fact that supplementary materials to this manuscript can be found at https://doi.org/10.6084/m9.figshare.19263629.v2.

## Supporting information



Supplementary Material
