# Supplementary material for: Changes in 24‐h energy expenditure, substrate oxidation, and body composition following resistance exercise and a high protein diet via whey protein supplementation in healthy older men
Source: Physiol Rep. 2022 Jun 15;10(11):e15268. doi: 10.14814/phy2.15268 (PMC9332127; doi:10.14814/phy2.15268)
Supplement: Supplementary file 1 — Supplementary Material [file PHY2-10-e15268-s001.docx]

**Supplementary data**

Table S1. Nutritional composition of the experimental supplements (per serving)^1^

| Component | Whey protein isolate (PRO)^1^  25 g | Control (CON)  23.75 g |
| --- | --- | --- |
| Energy (kcal) | 95 | 95 |
| Carbohydrate (g) | 0 | 23.75 |
| Protein (g) | 22.8 | 0 |
| *EAA (g)* | 11.1 | 0 |
| *Histidine (g)* | 0.5 | 0 |
| *Lysine (g)* | 2.2 | 0 |
| *Methionine (g)* | 0.4 | 0 |
| *Phenylalanine (g)* | 0.8 | 0 |
| *Threonine (g)* | 1.1 | 0 |
| *Tryptophan (g)* | 0.7 | 0 |
| *BCAA (g)* | 5.4 | 0 |
| *Leucine (g)* | 2.9 | 0 |
| *Isoleucine (g)* | 1.3 | 0 |
| *Valine (g)* | 1.2 | 0 |
| *NEAA (g)* | 11.7 | 0 |
| *Alanine (g)* | 1.0 | 0 |
| *Arginine (g)* | 0.5 | 0 |
| *Asparagine (g)* | 2.5 | 0 |
| *Cysteine (g)* | 0.6 | 0 |
| *Glutamine (g)* | 3.6 | 0 |
| *Glycine (g)* | 0.4 | 0 |
| *Proline (g)* | 1.1 | 0 |
| *Serine (g)* | 0.8 | 0 |
| *Tyrosine (g)* | 0.8 | 0 |
| Fat (g) | 0.4 | 0 |

^1^Whey protein isolate also contained per serving: vitamin A (<25 IU), vitamin C (<0.5 mg), vitamin D (0.2 μg), iron (0.25 mg), calcium (21.3 mg), phosphorus (85 mg), magnesium (2.5 mg) chloride (20 mg), sodium (172.5 mg), potassium (17.5 mg). BCAA, branched-chain amino acids; EAA, essential amino acids; NEAA, non-essential amino acids.

| **Meal**/Ingredients | **Contents, g** | **Carbohydrate, g** | **Protein, g** | **Fat, g** | **Energy, kcal** |
| --- | --- | --- | --- | --- | --- |
| **Evening meal (2000 h)** |  |  |  |  |  |
| Chicken tikka masala | 427 | 56.0 | 33.7 | 25.2 | 587 |
| **Breakfast (0915 h)** |  |  |  |  |  |
| Fruit and fibre cereal | 85 | 57.4 | 7.9 | 4.5 | 302 |
| Semi-skimmed milk | 156 | 7.5 | 5.6 | 2.8 | 78 |
| **Lunch (1240 h)** |  |  |  |  |  |
| Lasagne | 383 | 39.1 | 31.1 | 28.8 | 539 |
| Garlic bread | 57 | 28.2 | 2.9 | 9.5 | 209 |
| Whole milk yoghurt, Yeo valley strawberry | 125 | 13.1 | 12.0 | 4.7 | 143 |
| **Dinner (1720 h)** |  |  |  |  |  |
| Wholemeal bread | 119 | 45.0 | 12.6 | 3.3 | 261 |
| Margarine, Lurpack spreadable | 17 | 0.1 | 0.1 | 13.3 | 120 |
| Ham | 23 | 0.3 | 5.1 | 0.5 | 25 |
| Banana (without skin) | 136 | 31.6 | 1.6 | 0.4 | 136 |
|  |  | **Total, g** | | | **Total, kcal** |
|  |  | 279 | 112.6 | 92.9 | 2400 |
|  |  | **% energy intake** | | |  |
|  |  | 46% | 19% | 35% |  |

##### Table S2. Example chamber diet (ID: 029_baseline)^1^

^1^All products were purchased from Tesco, UK (<https://www.tesco.com/groceries/en-GB>).

**Non-exercise**

Analysed (n = 16)

♦ Excluded from analysis (n = 0)

Assessed for eligibility between October 2017 and February 2019 (n = 256)

Excluded (n = 217)

♦  Did not meet inclusion criteria (n = 132)

♦  Declined to participate (n = 85)

Randomised (n = 39)

Lost to follow-up
 (n = 2)

n = 1 withdrew during baseline chamber experiment

n = 1 withdrawn due to unrelated medical issue

Lost to follow-up
(n = 2)

n = 1 withdrew from chamber experiment prior to baseline

n = 1 withdrawn due to prescription of statins

Lost to follow-up
(n = 2)

n = 1 withdrew from chamber experiment prior to baseline

n = 1 withdrew due to time commitment

Lost to follow-up

(n = 0)

**RE**Analysed (n = 17)

♦ Excluded from analysis (n = 0)

Analysed (n = 8)

♦ Excluded from analysis (n = 0)

Analysed (n = 9)

♦ Excluded from analysis (n = 0)

Allocated to CON
(n = 10)

Allocated to PRO
(n = 10)

Allocated to RE+CON (n = 10)

Allocated to RE+PRO (n = 9)

Allocation

Follow-Up

Analysis

**CON**

Analysed (n = 16)

♦ Excluded from analysis (n = 0)

**PRO**

Analysed (n = 17)

♦ Excluded from analysis (n = 0)

Fig. S1 Study design and flow of participants throughout the study. Red lines = pooled groups for RE vs. non-exercise analysis; Green lines = pooled groups for PRO vs. CON analysis; Orange lines = RE+CON vs. RE+PRO analysis.

Fig. S2. Calculation of sedentary EE, activity EE (AEE_chamber_), spontaneous physical activity (SPA) and diet-induced thermogenesis (DIT) inside the respiration chamber using the intercept method for one participant (ID: 028_baseline). Energy expenditure was plotted against physical activity, both averaged over 30 min intervals, over the non-exercise intervals (21.5 h) of the 24-h measurement period. The *y*-intercept (*c*) represents EE at zero activity (EE_0_), defined as sedentary EE (kcal/min), which consists of sleeping metabolic rate (SMR) and DIT. AEE_chamber_ was calculated by subtracting sedentary EE from TEE, and the difference between sedentary EE and SMR represented DIT. The slope (*m*) of the regression line represents SPA (kcal/min per % activity), which was extrapolated to 24-h values (kcal/d per % activity) and multiplied by mean 24-h Activity_chamber_ to calculate SPA (kcal/d).

Table S3. Baseline characteristics of participants in the RE+CON and RE+PRO groups^1^

|  | RE+CON | RE+PRO | *P* value^3^ |
| --- | --- | --- | --- |
| *n* | 8 | 9 | - |
| Age, y | 67 ± 2 | 68 ± 1 | 0.48 |
| Height, m | 1.77 ± 0.02 | 1.74 ± 0.03 | 0.46 |
| Body mass, kg | 76.1 ± 3.8 | 80.9 ± 4.0 | 0.40 |
| BMI, kg/m^2^ | 24.4 ± 0.8 | 26.6 ± 0.8 | 0.08 |
| FFM, kg | 57.8 ± 2.8 | 60.5 ± 2.9 | 0.52 |
| SMM, kg | 25.6 ± 1.2 | 26.9 ± 1.3 | 0.48 |
| FM, kg | 18.2 ± 1.7 | 20.4 ± 1.5 | 0.35 |
| FM, % | 23.8 ± 1.6 | 25.1 ± 1.2 | 0.55 |
| TEE_chamber_, kcal/d | 2460 ± 86 | 2454 ± 86 | 0.97 |
| TEE_accelerometry_, kcal/d | 2639 ± 107 | 2605 ± 109 | 0.82 |
| RMR, kcal/d | 1631 ± 66 | 1609 ± 68 | 0.83 |
| PAL_chamber_ | 1.51 ± 0.05 | 1.53 ± 0.03 | 0.73 |
| PAL_accelerometry_ | 1.60 ± 0.03 | 1.57 ± 0.02 | 0.36 |
| Fasting plasma glucose, mmol/L | 5.9 ± 0.3 | 5.7 ± 0.2 | 0.58 |
| HOMA-IR | 2.3 ± 0.4 | 2.2 ± 0.4 | 0.90 |
| Step count, steps/d | 12,171 ± 1,153 | 11,346 ± 907 | 0.58 |
| Activity_chamber_, % | 17.1 ± 1.2 | 17.5 ± 0.9 | 0.79 |
| Activity_accelerometry_, counts/d | 366,047 ± 71,344 | 287,627 ± 46,739 | 0.36 |

^1^Values are means ± SE. ^3^*P* value refers to differences between groups analyzed by independent samples *t*-test. BMI, body mass index; FFM, fat-free mass; FM, fat mass; HOMA-IR, homeostatic model assessment of insulin resistance; SMM, skeletal muscle mass; PAL_accelerometry_, estimated physical activity level by accelerometry; PAL_chamber_, physical activity level calculated inside the respiration chamber; RMR, resting metabolic rate; TEE_accelerometry_, estimated total energy expenditure by accelerometry; TEE_chamber_, total energy expenditure calculated inside the respiration chamber.

##### Table S4. Self-report dietary intake during the intervention period in the RE+CON and RE+PRO groups^1^

|  | | RE+CON | | | RE+PRO | | |  |
| --- | --- | --- | --- | --- | --- | --- | --- | --- |
|  | | Baseline | 6 weeks | 12 weeks | Baseline | 6 weeks | 12 weeks | *P* value^2^ |
| Energy, kcal/d | |  |  |  |  |  |  |  |
| *Diet* | | 2071 ± 79 | 1972 ± 93 | 2021 ± 128 | 2080 ± 119 | 2048 ± 97 | 1969 ± 141 | 0.33 |
| *Total* | | 2071 ± 79 | 2163 ± 93 | 2211 ± 128 | 2080 ± 119 | 2238 ± 97^#^ | 2159 ± 141 | 0.30 |
| Protein, g/d | |  |  |  |  |  |  |  |
|  | *Diet* | 86 ± 6 | 86 ± 5 | 84 ± 5 | 81 ± 3 | 85 ± 6 | 79 ± 3 | 0.37 |
|  | *Total* | 86 ± 6 | 86 ± 5 | 84 ± 5 | 81 ± 3 | 131 ± 6^#*^ | 125 ± 3^#*^ | **< 0.001** |
| Protein, g/kg/d | |  |  |  |  |  |  |  |
|  | *Diet* | 1.13 ± 0.04 | 1.13 ± 0.05 | 1.09 ± 0.06 | 1.01 ± 0.06 | 1.07 ± 0.05 | 0.99 ± 0.05 | 0.92 |
|  | *Total* | 1.13 ± 0.04 | 1.13 ± 0.05 | 1.09 ± 0.06 | 1.01 ± 0.06 | 1.63 ± 0.07^#*^ | 1.58 ± 0.07^#*^ | **< 0.001** |
| Protein, % | |  |  |  |  |  |  |  |
|  | *Diet* | 16.5 ± 0.5 | 17.5 ± 0.9 | 16.9 ± 0.9 | 15.9 ± 0.7 | 16.6 ± 0.6 | 16.7 ± 1.1 | 0.76 |
|  | *Total* | 16.5 ± 0.5 | 15.9 ± 0.8 | 15.4 ± 0.8 | 15.9 ± 0.7 | 23.5 ± 0.6^#*^ | 23.8 ± 1.4^#*^ | **< 0.001** |
| Carbohydrate, g/d | |  |  |  |  |  |  |  |
|  | *Diet* | 233 ± 12 | 224 ± 16 | 231 ± 12 | 254 ± 16 | 221 ± 11 | 238 ± 16 | 0.78 |
|  | *Total* | 233 ± 12 | 272 ± 16^#*^ | 279 ± 12^#*^ | 254 ± 16 | 221 ± 11 | 238 ± 16 | **< 0.001** |
| Carbohydrate, % | |  |  |  |  |  |  |  |
|  | *Diet* | 45.3 ± 2.9 | 45.8 ± 1.9 | 46.4 ± 2.2 | 52.3 ± 3.8 | 43.5 ± 2.0 | 48.9 ± 2.4 | 0.23 |
|  | *Total* | 45.3 ± 2.9 | 50.3 ± 2.0* | 51.0 ± 2.2^#*^ | 52.3 ± 3.8 | 39.7 ± 1.8 | 44.4 ± 2.1 | **< 0.001** |
| Fat, g/d | |  |  |  |  |  |  |  |
|  | *Diet* | 76 ± 7 | 71 ± 6 | 70 ± 9 | 71 ± 6 | 77 ± 5 | 61 ± 8 | 0.25 |
|  | *Total* | 76 ± 7 | 71 ± 6 | 70 ± 9 | 71 ± 6 | 78 ± 5 | 62 ± 8 | 0.24 |
| Fat, % | |  |  |  |  |  |  |  |
|  | *Diet* | 32.7 ± 2.5 | 32.0 ± 1.7 | 30.5 ± 2.5 | 30.7 ± 2.2 | 33.7 ± 1.1 | 27.4 ± 1.8 | 0.24 |
|  | *Total* | 32.7 ± 2.5 | 29.2 ± 1.6 | 27.9 ± 2.4 | 30.7 ± 2.2 | 31.1 ± 1.1 | 25.3 ± 1.7 | 0.24 |

^1^Values are means ± SE.  ^2^*P* value refers to respective group-by-time interaction. Diet = intake from habitual intake (excluding experimental supplements). Total = intake from habitual diet plus experimental supplements. ^#^*P* < 0.05 from baseline value. *Significant difference between groups at respective time point.

Table S5. Energy expenditure and 24-h macronutrient oxidation and balances for the RE+CON and RE+PRO groups at baseline and 12 weeks^1^

|  | RE+CON | | RE+PRO | |  |  |
| --- | --- | --- | --- | --- | --- | --- |
|  | Baseline | 12 weeks | Baseline | 12 weeks | *P* value^2^ | |
| TEE_chamber_, kcal/d | 2460 ± 86 | 2413 ± 96 | 2455 ± 86 | 2439 ± 88 | 0.32 | |
| TEE_accelerometry_, kcal/d | 2639 ± 107 | 2635 ± 101 | 2605 ± 109 | 2591 ± 102 | 0.80 | |
| Sedentary EE, kcal/d | 1711 ± 47 | 1750 ± 75 | 1720 ± 75 | 1798 ± 64 | 0.55 | |
| RMR, kcal/d | 1631 ± 66 | 1670 ± 64 | 1609 ± 68 | 1643 ± 65 | 0.80 | |
| SMR,^3^ kcal/d | 1562 ± 79 | 1607 ± 78^#^ | 1598 ± 64 | 1643 ± 60^#^ | 0.90 | |
| AEE_chamber_, kcal/d | 748 ± 59 | 663 ± 30 | 735 ± 47 | 642 ± 41 | 0.73 | |
| AEE_accelerometry_, kcal/d | 867 ± 75 | 793 ± 56^#^ | 784 ± 49 | 678 ± 30^#^ | 0.10 | |
| SPA, kcal/d | 421 ± 43 | 345 ± 16 | 407 ± 46 | 297 ± 33^#^ | 0.27 | |
| DIT, kcal/d | 153 ± 31 | 170 ± 20 | 122 ± 38 | 155 ± 25 | 0.58 | |
| DIT, % of EI | 6.7 ± 1.4 | 7.1 ± 0.9 | 4.9 ± 1.5 | 6.4 ± 1.1 | 0.63 | |
| PAL_chamber_ | 1.51 ± 0.05 | 1.45 ± 0.05^#^ | 1.53 ± 0.03 | 1.49 ± 0.03 | 0.46 | |
| PAL_accelerometry_ | 1.60 ± 0.03 | 1.57 ± 0.02^#^ | 1.57 ± 0.02 | 1.53 ± 0.01^#^ | 0.10 | |
| Protein oxidation, g/d | 84 ± 6 | 80 ± 6 | 83 ± 6 | 101 ± 7 | **0.01** | |
| Carbohydrate oxidation, g/d | 240 ± 14 | 252 ± 14 | 240 ± 16 | 245 ± 7 | 0.89 | |
| Fat oxidation, g/d | 110 ± 7 | 106 ± 5 | 105 ± 6 | 97 ± 7 | 0.45 | |
| RQ | 0.84 ± 0.01 | 0.84 ± 0.01 | 0.84 ± 0.01 | 0.84 ± 0.01 | 0.65 | |
| Protein balance, g/d | 31 ± 6 | 36 ± 4 | 28 ± 4 | 9 ± 5^#^ | **< 0.001** | |
| Carbohydrate balance, g/d | 56 ± 11 | 48 ± 9 | 41 ± 11 | 55 ± 12 | 0.69 | |
| Fat balance, g/d | -15 ± 9 | -9 ± 6 | -9 ± 9 | -1 ± 7 | 0.44 | |
| EB_chamber_, kcal/d | 44 ± 98 | 73 ± 74 | 37 ± 61 | 61 ± 37 | 0.31 | |
| EB_free-living_, kcal/d | -568 ± 75 | -423 ± 86^#^ | -525 ± 110 | -433 ± 122 | 0.52 | |

^1^Values are means ± SE. Energy expenditure and substrate oxidation values are reported unadjusted. ^2^*P* value refers to respective group-by-time interaction. ^3^RE+CON; *n* = 7 (*n* = 1 outlier >3SD from mean removed from analysis as described in the main text). AEE_accelerometry_, estimated activity energy expenditure by accelerometry; AEE_chamber_, activity energy expenditure calculated inside the respiration chamber; DIT, diet-induced thermogenesis; EB_chamber_, calculated energy balance inside the respiration chamber; EB_free-living_, estimated energy balance in free-living; PAL_accelerometry_, estimated physical activity level by accelerometry; PAL_chamber_, physical activity level calculated inside the respiration chamber; RMR, resting metabolic rate; RQ, respiratory quotient; SMR, sleeping metabolic rate; SPA, spontaneous physical activity; TEE_accelerometry_, estimated total energy expenditure by accelerometry; TEE_chamber_, total energy expenditure calculated inside the respiration chamber. ^#^*P* < 0.05 from baseline value.

Table S6. Step exercise energy expenditure and substrate oxidation for the RE+CON and RE+PRO groups at baseline and 12 weeks^1^

|  | RE+CON | | RE+PRO | |  |  |
| --- | --- | --- | --- | --- | --- | --- |
|  | Baseline | 12 weeks | Baseline | 12 weeks | *P* value^2^ | |
| **EX-1** |  |  |  |  |  | |
| EE, kcal/min | 4.7 ± 0.2 | 4.6 ± 0.2 | 4.7 ± 0.2 | 4.8 ± 0.2 | 0.18 | |
| Carbohydrate oxidation, g/d | 693 ± 68 | 688 ± 54 | 760 ± 47 | 776 ± 47 | 0.38 | |
| Fat oxidation, g/d | 351 ± 24 | 342 ± 22 | 328 ± 25 | 326 ± 26 | >0.99 | |
| Protein oxidation, g/d | 85 ± 9 | 85 ± 8 | 88 ± 7 | 92 ± 9 | 0.62 | |
| RQ | 0.84 ± 0.01 | 0.84 ± 0.01 | 0.85 ± 0.01 | 0.85 ± 0.01 | 0.53 | |
| **EX-2** |  |  |  |  |  | |
| EE, kcal/min | 4.8 ± 0.2 | 4.7 ± 0.3^#^ | 4.9 ± 0.2 | 5.0 ± 0.2 | 0.09 | |
| Carbohydrate oxidation, g/d | 1143 ± 80 | 1091 ± 68 | 1154 ± 58 | 1168 ± 54 | 0.10 | |
| Fat oxidation, g/d | 174 ± 20 | 191 ± 17 | 173 ± 12 | 190 ± 24 | >0.99 | |
| Protein oxidation, g/d | 85 ± 9 | 85 ± 8 | 88 ± 7 | 92 ± 9 | 0.62 | |
| RQ | 0.92 ± 0.01 | 0.91 ± 0.01 | 0.92 ± 0.01 | 0.91 ± 0.01 | >0.99 | |
| **EX-3** |  |  |  |  |  | |
| EE, kcal/min | 4.9 ± 0.2 | 4.7 ± 0.3^#^ | 4.9 ± 0.2 | 5.0 ± 0.2 | 0.11 | |
| Carbohydrate oxidation, g/d | 1133 ± 71 | 1152 ± 64 | 1172 ± 53 | 1219 ± 56 | 0.53 | |
| Fat oxidation, g/d | 179 ± 24 | 162 ± 19 | 170 ± 13 | 168 ± 14 | 0.46 | |
| Protein oxidation, g/d | 85 ± 9 | 85 ± 8 | 88 ± 7 | 92 ± 9 | 0.62 | |
| RQ | 0.92 ± 0.01 | 0.92 ± 0.01 | 0.91 ± 0.01 | 0.92 ± 0.01 | 0.91 | |

^1^Values are means ± SE. Energy expenditure and substrate oxidation values are reported unadjusted. ^2^*P* value refers to respective group-by-time interaction. All step exercise bouts were performed at a step rate of 75 steps/min for 30 min. Protein oxidation data has been added for completeness but was obtained from one urine sample between 0800-2000 h. EE, energy expenditure; EX-1, step exercise bout 1 (0830 h in the fasted state); EX-2, step exercise bout 2 (1445 h); EX-3, step exercise bout 3 (1915 h). ^#^*P* < 0.05 from baseline value.
